# Toxicities that matter: Slow bureaucracy and polluting temporalities in a southern Italian city

**DOI:** 10.1177/23996544221107517

**Published:** 2022-06-24

**Authors:** A Raffaele Ippolito

**Affiliations:** School of Geography and the Environment, RinggolID: 6396 University of Oxford, Oxford, UK

**Keywords:** pollution, Taranto, ethnography, slow violence, environmental justice

## Abstract

This paper deconstructs toxicity through a juxtaposition of a conventional epidemiological approach to pollutants and the lived experience of a highly polluted residential area next to the largest steel production plant in Europe. An ethnographic analysis of toxicity in Taranto illustrates the complexity of various temporal scales through which toxic chemicals contribute to new biological, political and moral balances. Attuning to the slow experiences of pollution is fundamental to shed light on the processes moulding aspirations to environmental justice within the community. In particular, law and bureaucracy imbue pollutants with experiential legitimacy, allowing them to be seen, contested and collectivized. I focus on the residents’ and workers’ dissonant experiences with the bureaucratic system to illustrate how their encounter with asbestos has profoundly shaped toxic exposure to less bureaucratically visible shapeshifting pollutants, thus contributing to a diffused sense of political resignation.

## Introduction

The flats in the *Tamburi* district are not like other buildings in Taranto, they are brown and red on the outside, and if one looks closely, they will realize their walls are stained with a thick layer of dust, mostly red iron oxide. The *‘minerale’*, as people call it in Taranto, is only part of the complex cocktail of chemicals that results in a higher incidence and prevalence of cancer, cardiovascular and respiratory diseases in the neighborhood than the national average (Banini and Palagiano, 2014). Adjacent to this working-class district, less than 200 m away from the first block of council flats, there are giant walls. Inside them, there is an industrial park that provides work for over 14,000 people in the city, many of whom live in *Tamburi*. Residents have many names for the industrial park, in particular for the largest of its industries. They call it ex-Ilva, the Factory, the Plant, the Monster. Currently co-managed by a state-owned enterprise and the ArcelorMittal group, ex-Ilva is the largest steel mill in Europe. It opened in 1961 as part of a wider national development plan which also aimed at creating new jobs and promoting economic development in the south of Italy (Pennuzzi, 2001). After being privatized in 1995, it was sequestrated in 2012 over accusations of manslaughter due to illegal polluting activities. After 5 years under special administration, it was acquired by the ArcelorMittal group in 2018. Over the decades, Ex-Ilva has become the landmark of Taranto and stands as a powerful symbol of the Italian heavy industry.

Today, a petrochemical refinery stands next to ex-Ilva, together with an industrial landfill and an industrial port. Despite the efforts of a few local environmentalists to put an end to the problem, toxic substances have persisted in high concentrations for over 50 years. Epidemiological investigations spanning over two decades have linked industrial pollution to the high rates of mortality and morbidity among Taranto’s residents, a result of various forms of cancer, respiratory and cardiovascular diseases (Mangia et al., 2013; Mataloni et al., 2012; Pirastu et al., 2010). Nevertheless, this evidence has not spurred regional and national institutions and most residents to take action – on the contrary, many seem to have resigned to living with pollution and its impacts and are reluctant to speak out against the factory (Ippolito, 2022). Ethnographies of environmental injustice have often told the stories of communities that claim harm due to toxic exposures but are unable to prove it through scientific evidence (see Brown, 2007; Lerner, 2005). However, the experience of some residents of Taranto seems to be the opposite case, with consistent evidence but weak advocacy for change among the general population. What then originates this disconnect between epidemiological evidence, institutional and community action?

Understanding these discrepancies requires acknowledging the historical premises on which industrialization took place in Taranto. Southern Italy was historically excluded from the process of industrialization that took place in the north of the country in the first half of the 20^th^ century (Gomellini and Toniolo, 2017). The high levels of unemployment resulting from this condition granted the fewer industrial groups that were later established in the south greater power to resist demands for environmental justice (Barca and Leonardi, 2018). These conditions reproduced a form of structural violence that blackmailed residents economically and portrayed them as complicit in the public health disaster associated with pollution (Greco, 2016).

The systemic suppression of community voices at the hands of business and institutional actors has also prevented the making of a class-based environmental consciousness (Barca and Leonardi, 2016). In a few cases, the workers’ attempts to theorize and explain the social implications of industrialization and pollution have not been integrated within more academically established environmental justice frameworks. Lorenzin and Devi (2021) build on this notion to argue how in the environmental justice struggles of the Porto Marghera area – despite the involvement of some prominent intellectuals such as Antonio Negri in the working-class movement – the voices of workers remained unheard, rather than unspoken. Instead of a lack of public participation, the issue at stake can be considered a mismatch of everyday epistemologies, systems of valuation and conceptualizations of agency between residents, grassroots organizations, institutions and academia.

Building on the above premises, this paper unpacks some of the nuances and tensions that shape Taranto’s experience of pollution and illustrates how they contribute to a vernacular reframing of notions of toxic exposures and environmental justice. Rather than attempting to provide an analysis of the issue of political resignation, I reflect on the role of pollutants as political actors that also shape the experiential frames of toxic exposures, resulting in a misalignment with an epidemiological approach to toxicity. This understanding of pollutants acknowledges their phenomenological entanglements within the dimensions of law, bureaucracy and culture, which operate through different temporal scales. Thus, I disentangle the experiential contradictions of pollutants to draw attention to the extent to which their dissonant temporalities produce narratives that are connected to the community’s resignation to pollution.

The data analyzed in this article were collected through ethnographic fieldwork carried out in *Tamburi,* the city’s most polluted working-class neighborhood (Mangia et al., 2013), between 2017 and 2020. Participant observation conducted within a 12-month period took place through daily interactions and participation in meetings with residents, factory workers, environmental activists and medical and legal professionals. This was complemented with 47 semi-structured in-depth interviews with key opinion leaders and residents that provided either technical information or in-depth reflections of daily encounters with pollution.

Being originally from Taranto myself and having lived in the city for most of my life, I recruited informants through my own informal networks and long-term acquaintances. Out of all participants, nine were friends and family members with whom I have cultivated a personal relationship for years. My positionality in the field constituted an advantage in this type of research, given that my study of Taranto is informed by a lived experience of the place that goes well beyond the timeframe and scope of this paper. Such temporally diluted participation in the everyday social life of pollution and the community allowed for a study of the slow experiences that shape and are shaped by toxic flows, shedding light on the subtle politics of chemical exposure that greatly affect how people in Taranto relate to their environment.

The experiences and views of informants were heterogeneous and reflected different socio-economic backgrounds. Residents of Tamburi and factory workers were less likely to participate in initiatives organized by advocacy groups, while some of the activists themselves were legal experts, medical professionals, teachers and business owners. This class divide had important implications for the type of evidence informing understandings and experiences of pollution. Activists’ valued scientific evidence and information recorded in official documents: they based their claims on the latest epidemiological reports, court proceedings, investigations and data they collected themselves through air monitoring devices. On the other hand, residents and workers drew heavily on their daily observations on pollution, recalling life events and embodied encounters with the toxic to articulate their claims. They would focus on the smells, the mineral dust they swept off their balconies on a given day, the stains on the laundry left to dry in the sun and the worry of waiting for the results of a medical examination. This diversity of data produced diverse understandings of pollution and aspirations to the environment and partly explained the apparent lack of engagement of Tamburi residents in environmental advocacy groups.

In this paper, I draw on the two sets of experiences described above to highlight the fundamental importance of the contingent in molding environmental subjectivities. Nicholas Shapiro (2015) describes the imperceptible encounters between the chemical and the human worlds where bodily accumulations of pollutants are diluted in time and space and emerge only through lived experience. Through the notion of *‘chemical sublime’* he describes a process in which the exposed make sense of their narratives of contamination over extended periods of time, eventually leading to political consideration and potential intervention. In order to understand how communities mobilize and pursue environmental justice, it is foremost necessary to understand the intimate meaning they attach to the slow environmental and bodily changes happening around/within them. Thom Davies (2018) names this practice *slow observation* and uses it to call attention to the knowledge on environmental degradation that communities can provide. To neglect slow observation would mean to reinforce a form of epistemic violence that would lead to further marginalization and environmental suffering (Davies, 2019). *Slow observation* is echoed and supported by pleas for *slow science* (Stengers, 2018), *slow scholarship* (Mountz et al., 2015) and *slowing down* (Whatmore, 2009: Bingham, 2008)*.*

A slow approach to environmental injustice can strip environmental science of its neoliberal assumptions on what ‘counts’ as evidence (Waldman, 2009) and allows for a deeper theoretical integration with the knowledge practices of the communities at stake. Slow observation can allow for a re-interpretation of that silence in what Hatzisavvidou (2015) describes as a practice aimed at the generation of new political subjectivities and the reconfiguration of pre-existing power relations. In doing so, slow observation can question the very assumptions on the notion of ‘silence’ operating within environmental justice, moving beyond narratives of political apathy or superimposed immobility (Barnett, 2016) into new explorations of how silence might be practiced and mobilized democratically (Jungkunz, 2012).

Following the notion of *slow observation*, I attune to Taranto’s micro experiences of bureaucratic practices built around pollution. I argue that, despite the growing body of epidemiological evidence correlating disease and pollution, the legal framework around some pollutants has been negotiated, contested, made and unmade over time, attributing to said pollutants blurred narratives that residents cannot fully experience. In the first part of the article, through the exposure experience of dioxin and benzo(a)pyrene, I suggest an understanding of some chemicals as *shapeshifting pollutants* with political properties that frame and reframe toxicity through their interaction with the legal system. Over time, these continuous changes are responsible for their detachment from the lived experience of residents.

The term ‘shapeshifting’ is a lay reference to the work of Bismillah et al. (2020) on a class of molecules in which atoms are re-arranged in response to specific environments. Although the pollutants considered in this article do not belong to that class of molecules from a chemical standpoint, I borrow the notion to emphasize the transcendentality of shapeshifting properties into the political dimension of substances. Shapeshifting can also entail legal and bureaucratic structures that change according to the political environment in which they are enacted.

In the second part of the article, to better elucidate the modalities through which dioxin, benzo(a)pyrene and other pollutants *shapeshift* through fluctuations in the legal system, I contrast these to the workers’ exposure to a *non-shapeshifting* pollutant – asbestos. The political shape of asbestos was crystallized in Italian law and bureaucracy in the first half of the 1990s, hence delineating a clear narrative that workers have lived and experienced for two generations, up until 2003. Given asbestos’ experiential monopoly on the legal and bureaucratic life of exposure, this pollutant is key to understanding some of the resident’s attitudes to other toxic substances that are less regulated and more epidemiologically relevant today. I conclude that the delayed temporality of some asbestos-related diseases and their recognition in Italian law result in an illness paradigm that calibrates how workers experience exposure to other toxic substances. This subsequently contributes to a perception of injustice as irremediable, but also allows workers and residents to engage with a type of observation that leads them to reconfigure environmental justice over time.

### Making sense of shapeshifting pollutants

The industrial park of Taranto emitted over 11,000 tons of nitrogen dioxide and sulfur dioxide in 2010 alone, breaching the limit values imposed by national legislation (Vulpio, 2012). The industrial park is responsible for releasing a toxic cocktail into the air of Taranto, which also includes particulate matter (PM), polycyclic aromatic hydrocarbons (PAH) and dioxin (Pirastu et al., 2010). Epidemiological surveys have highlighted excess mortality in the areas adjacent to the industrial zone (Vigotti et al., 2014). However, despite this growing body of epidemiological evidence, Italian legislation lacks a framework that recognizes the causality between pollutants and pathologies, mostly because this is based on probabilistic information (Checker, 2007). Thus, Taranto’s excess of morbidity and mortality for pathologies correlated to industrial pollutants cannot be contested through specific legal procedures that establish liability for said health effects (Barca and Leonardi, 2016).

The legal ambiguity of pollutants overlaps with a lack of well-maintained environmental and public health data repositories in Taranto. The concerned citizens, lawyers, journalists and medical professionals interviewed reported a general fragmentation of the data available to the public, which resulted in struggles to access information. The Regional Agency for Environmental Protection and Prevention (ARPA) is here taken as an example. ARPA oversees the collection and monitoring of the data on pollution on its website and makes it available for a wide range of scopes. A local journalist elucidated the situation:

ARPA doesn’t update its website well enough, the data collected overlooks so many variables and is not exhaustively explanatory. In the last years it got even worse because the new director is a lawyer and [I believe] has been appointed for political reasons. It was so difficult to get to talk to him – he would refuse to release interviews - even just over the phone. […] So, in a place like Taranto, in which some journalists and residents are hungry for data, we cannot rely on ARPA as a reliable source of information.

The assumption that some public officials are complicit to the factory is a widespread belief among Taranto’s environmental activists. A medical professional similarly recalled that the director of a local public health institute once opposed a study on children’s pathologies correlated to industrial pollution they had been campaigning for, arguably for political purposes. At the grassroots level, this creates what Gullion (2015) calls *structural betrayals,* in which the community seeks help from local agencies but fails to obtain it. As the term suggests, *structural betrayals* do not exclusively depend on individual officials’ unwillingness to help but can be also attributed to bureaucratic mechanisms that prevent them from granting access to information (Gullion, 2015).

The dissipation of institutional support to activists is thus enacted through a dilution of individual authority in bureaucratic processes. This leads to a discouragement of the public in pursuing environmental action and in a diffused mistrust of public authority. In this climate of bureaucratic uncertainty, in which no one can be trusted, information also becomes highly politicized. Thus, since data are generated through political interaction, they are no longer objective, and, as such, can always be contested a priori. When some interviewed activists carried out an analysis on locally farmed mussels which revealed dangerously high concentrations of dioxins, they were threatened and had to shield at home for months. The intimidations came from mussel farmers and residents in the districts closest to the factory, who accused them of pushing forward their personal agenda to rehabilitate their reputation as environmentalists. In the same way, the monitoring of data the factory produces on its own emissions is automatically regarded as incorrect by environmental activists, given the countless scandals in which official reports were contradicted by external investigations.

The lack of a strong institutional framework, the political contestation of data from multiple actors and the bureaucratic uncertainty experienced by the community are amplified by the presence of special laws – often referred to as *Decreti Salva-Ilva* (*Save-Ilva decrees)* and implemented from 2010 to 2020 – aiming at ‘saving’ the industry from financial disaster and mitigating the financial debt accumulated over the years. Among the measures adopted, the thresholds for some pollutants which were extremely high in Taranto have been suspended nationally. Between 2008 and 2010, alarmed by new data on benzo(a)pyrene – an infamous carcinogenic pollutant – a few environmental organizations in Taranto campaigned for the regional authorities to begin an official monitoring of its concentrations, which was eventually commissioned by the regional government in 2010. Some estimations made by activists and independent researchers showed that the emissions of the pollutant would be above the legal thresholds. A few months before the official monitoring started, the Italian government passed a law which suspended the legal thresholds of the pollutant. The results of the monitoring carried out by the regional authority therefore did not highlight any abnormal activity. As described above, the mutability of the meanings attributed to pollution data via law resulted in a profound sense of defeat in the community. Alessandro Marescotti, one of the key environmentalists in Taranto, recalled:

In November 2008 there was a protest with 20,000 people. Less than two years later, when they made the first Salva-Ilva law, the same amount of people joined a protest. From that moment on, the government took a very hard stance on the issue. They sent the message that we could be as many as we wanted, that we could even bring 200,000 people to protest, they would simply annihilate whatever protest law after law. They – the people in power – want to send the message that no matter what action you take, they can change the rules.

The making and unmaking of the thresholds that define the acceptability of pollutants imply that the very biological ‘autonomy’ of the community is contingent upon a set of external norms (Petryna, 2013) and is connected to the widespread sense of political resignation to pollution among workers. Within this context, the workers perceive a disconnect between epidemiology and action for environmental health and feel that community action on environmental injustice can be overridden by ad-hoc laws (Ippolito, 2022).

Over the course of fieldwork, this became an issue that I extensively discussed with Alessandro. He invested his energies into organizing and analyzing the data produced by environmental and public health agencies to make it accessible to the lawyers working on one of the largest trials for environmental disaster in Italian history, which also involved accusations of food poisoning, corruption and manslaughter.^1^ Despite maintaining that the disconnect between institutions and epidemiology is responsible for the weak participation on behalf of the community in the trial, he also added that local culture reflects the perceived vagueness of epidemiological evidence and institutional processes. We discussed this in relation to residents buying mussels – a well-known culinary symbol of Taranto – that were famously polluted with high concentrations of dioxin. He said:

Then you have those who are like ‘I don’t care’, they go there [to the fish market], they take responsibility, and say: ‘give me a kilogram of dioxin - I want my kilogram of mussels’. […] I think we also need to think about what value people attribute to their own life. It’s the same thing as taking a packet of cigarettes and reading ‘smoking kills’ but then you smoke anyways. In the same way, people eat mussels because they taste good, just like the people who smoke cigarettes. You could even sell them in bags that say ‘mussels kill’ - they’d still eat them anyways. The idea is that if something hurts you tomorrow then you are careful, but if it hurts you in 10 years, then ‘whatever, it’s 10 years!’

The issue raised by Alessandro is key to understanding the experience of pollution in Taranto. The sense of delayed toxicity he mentions evokes the notion of *slow violence*, a temporally diluted form of harm that dissipates the urgency for prevention and reaction to pollution (Nixon, 2011). Other ethnographies have described how *slow violence* leads to the naturalization of pollution in some communities, with residents accepting it as a normal component of everyday life (Auyero and Swistun, 2009; Lora-Wainwright, 2017). This amplifies the sense of resignation in the residents, which is reinforced by the delegitimization of the data available through unresponsive institutions and slow bureaucracy, an issue that has also been the object of ethnographic scrutiny (Auyero and Swistun, 2009; Fortun, 2001; Gullion, 2015). Over the years, the systemic recoding of pollutants has led to a protean discourse of toxicity as something institutionally negotiated and beyond the reach of collective action. From this perspective, *slow violence* in Taranto is also perpetrated via the mutable legal and bureaucratic configuration of the *shapeshifting pollutants* in its air, which prevents the emergence of a narrative of exposure that the community can experience and talk about. This lack of a fixed vocabulary to address the legal and bureaucratic properties of some pollutants is precisely what turns them into what an attorney described a ‘messy toxic cocktail’ in which correlations and liabilities are inextricably entangled.

The slow temporal dimension of toxicity in Taranto therefore intersects with the legal characterization of pollution. Mah and Davies (2020) draw attention to the simultaneous existence of multiple truths about toxicity that compete for historical, scientific and legal validation. In an attempt to break the conventional narrative that places scientific evidence at the top of the hierarchy of truths in Taranto’s struggle with pollution, a turn to *slow observation* is required to attune to the intimate experience of pollutants in the community. The mode of *slow observation* stretches the temporalities of pollution beyond the ‘now’ of an environmental health approach and allows to retrace toxic flows from a past that has contributed to today’s perception of toxicity. Below I focus on another toxic experience that has contributed to the community’s attunement to the slow dimension of law and bureaucracy: asbestos exposure.

### Attuning to bureaucratic temporalities

Given its almost unequivocal correlation to asbestosis and pleural mesothelioma, asbestos is a toxic substance whose adverse health effects have complete legal recognition in Italy (Laws 455/1993 and 232/2016, D.P.R. 336/1994). Those affected have thereby gained access to a compensation system that creates entitlements to early retirement upon the diagnosis of illness as well as direct monetary compensation (Paglietti et al., 2012). Asbestos is, for this reason, the only pollutant that has widely penetrated the social commentary on the environmental situation in Taranto, allowing workers to add a well-defined bureaucratic dimension to their experience of contamination. Even though asbestos is no longer actively utilized since 1992, over 3750 unremoved tons of the pollutant are reportedly still present in Taranto’s industrial park (Congedo, 2017). With exposure being no longer direct, asbestos continues to render the factory a potential risk, which concerns all residents irrespectively of their employment within the factory.

Asbestos as an occupational risk directly affected the generations of Ilva workers who entered the factory before 2003.^2^ On the other hand, the post-2003 workers did not experience asbestos as an element of direct exposure. However, through the installation of a compensation system and the high number of ex-Ilva workers that gained access to compensation payments, the pollutant has penetrated social discourse within and beyond the factory until today, adding a legal narrative of contamination for all workers and residents of the districts surrounding the industrial zone. It is necessary to note that, although some pathologies (especially pleural mesothelioma and asbestosis) have a strong scientific correlation to asbestos, their recognition in law is not premised on stronger evidence than that linking dioxin and benzo(a)pyrene to other pathologies. An attorney in Taranto interviewed in 2018 explained this in relation to the ongoing trial mentioned above:

The scientific literature available on asbestos wasn’t significantly more extensive than what we have on other pollutants in Taranto. Legislators have taken a series of measures in assuming that asbestosis and asbestos are connected. As a result, the patients affected by this illness and the workers have benefitted from a series of advantages. The same thing has not happened with dioxin, for example. We are now awaiting the verdict of the Senior Criminal Court regarding dioxin contamination [in the ongoing trial]. It might be that the judges will condemn the defendants and determine that they have to compensate the plaintiffs. However, given that no criminal judge can determine the damage, they might forward the matter to a civil judge, so that they can quantify the damage. At that point, it is likely that the civil judge will ask for an etiological proof, in which case we will have to collect more evidence on the correlation between exposure and illness. This procedure could take years.

This strong legal and political framework that connects asbestos to illness and economic compensation has profound implications in the ways workers understand pollution and contamination of other toxic substances. Experiencing asbestos and becoming ill comes with a bureaucratic experience of specific therapeutic patterns, as well as a compensation system that involves a bureaucratically mediated litigation between the factory and the exposed. Given its institutionally structured political narrative, asbestos is not a *shapeshifting pollutant*. For this reason, its experience has regulated the approach to toxicity of a wider conglomerate of *shapeshifting pollutants* that lack linear illness narratives and claim-making procedures.

The workers’ experience of asbestos transforms Taranto’s understanding of other toxic substances. Many of those who worked with asbestos recall exposure as a bureaucratic – rather than toxic – experience and, as such, cannot fathom exposure to other pollutants as they lack this dimension. The retired workers interviewed possess extensive knowledge of asbestos, the ways through which they had been exposed and the bureaucratic practices to recognize exposure. This knowledge of the pollutant was developed through the process of having exposure recognized formally to access compensation. A retired worker explained:

Back then people could retire thanks to asbestos. They could apply to the early retirement scheme. They [the management] would waive them a certain number of years of tax contributions so that they could go home early. Thanks to asbestos some people even retired at the age of 47! […] In my case, even though I wasn’t really in touch with asbestos, in the end I was recognized as exposed. I was lucky: I didn’t work asbestos, but in a way, I was exposed!

In cases like this one, exposure to asbestos is completely detached from the experience of harm: this particular worker recalled exposure as a positive event in his career, which allowed him to retire at the age of 53. Whilst the scientific evidence on asbestos has been validated through national legislation and is widely acknowledged by the population, it only informs bureaucratic action and can be experientially detached from exposure and illness.

Another aspect of asbestos exposure that contributed to the workers’ general experience of toxicity is the temporal dimension of exposure. Given that workers have been exposed to the pollutant over long periods of time, and that asbestos-related diseases are characterized by extended latency periods, some of the workers cannot relate their experience of exposure to that of illness (Mazzeo, 2018). The temporal disconnect between these two events implies an understanding of illness as something that can occur any time. This unpredictability of disease onset is paired with the short-life expectancy of patients with a mesothelioma diagnosis, which averages between 15 and 22 months (Pass et al., 2016). Therefore, asbestos-related disease is locally understood as ‘a time bomb that can explode at any given time’, as one worker put it. The lack of preventive measures on the workplace, paired with long latency periods of asbestos-related diseases and a short-life expectancy, imply the widespread belief that illness can occur any time regardless of the workers’ efforts to minimize exposure. Workers’ use of asbestos over prolonged periods of time is therefore paired with extended temporal gaps between exposure and onset of illness. Given these delayed temporalities of asbestos, the workers cannot easily inscribe exposure within the realm of the toxic, hence recollecting it as a bureaucratic practice that is enacted retrospectively.

More interestingly, when illness occurs, the experience of injustice and the pursuit of justice are also articulated through the bureaucratic practices that involve recognizing correlation to asbestos exposure and claiming for compensation. Under Italian law, the diagnosis of an occupational illness entitles the worker to be recognized as disabled first, and then apply for compensation via the National Institute of Insurance of Occupational Injury (INAIL) (National Asbestos Observatory, 2018). Among Taranto’s workers, it is also a standard practice to sue the employer and request further compensation, to which usually follows the offer of a settlement payment. The family of the worker can then decide whether to take up the settlement or continue with the lawsuit. Maria, the widow of a worker that died to pleural mesothelioma, recalled her experience with the compensation system:

When we eventually got the answer from INAIL, it said that he was recognized 100% of the damage, and they would proceed to calculate the amount of compensation and get back to us. When the check with the money eventually arrived, I burst into tears because I didn’t want that money. Every time we would go and talk to them [the INAIL officers] and they didn’t know what to say, I felt a sense of defeat, because I told myself ‘it’s true, then. He’s really dying’. It was something about my consciousness. When the money eventually arrived, I cried, hugged him and he said: ‘what are you crying for? Even if they don’t give us this money, my illness is not going away’. […] To me that money was dirty, I didn’t want it, I cried. I wanted his health. You get it?

In this case, the compensation received was the official evidence of the correlation between asbestos exposure and illness. Another informant described it as ‘the confirmation that death is really happening’, also meaning that the bureaucratic narrative on asbestos that was only potentially going to affect life is now being fulfilled. While this bureaucratic experience acknowledges biological decay, it also allows individuals to reframe illness as injustice, constituting a language granting legitimacy to one’s suffering. The ‘sense of defeat’ perceived by Maria was the result of a ‘cognitive’ epiphany that brought the plot of exposure and death to life and allowed it to be seen as the necropower (Mbembe, 2019; Davies, 2018) exercised on the workers at the hands of the factory and bureaucracy. This act carries an ontological power over injustice, bringing it into the local moral world (Kleinman, 2008) of the sufferers and allowing it to be lived.

The bureaucratic recognition of causality also entitles individuals to act on the injustice within the predetermined frames of action set up by law. Following the death of Maria’s husband, his family decided to sue Ilva. Maria’s daughter was confident that they would win the lawsuit and receive a higher compensation from the factory. However, they were later approached by the ex-Ilva’s legal representatives who asked them to settle for a lower amount than the one they asked. They refused the offer and continued with the lawsuit. When I asked her about her personal understanding of the compensation in relation to her father’s death, she said:

When we talked about it [whether accepting the settlement or not], we decided to look at the situation from a different perspective. It is not about the compensation, but about the possibility that there on the paper we can read: ‘this time they did not win.’ To me, going to bed at night and thinking ‘my family came to a compromise with Ilva’ or ‘Ilva has won over this family’ would equal losing my father ten times.

The bureaucratization of exposure creates pathways for action, granting individuals some agency in choosing the approach to pursue justice. Within this framework, demanding compensation is a tool to exercise power over the factory and access justice. When the workers’ families fail to prove correlation to exposure, they are excluded from making justice via bureaucracy. This causes another type of disempowerment, one that is illegitimate, and which cannot be articulated. Another woman lost her husband to lung cancer (as opposed to mesothelioma) but could not apply for compensation because his disease was not recognized as occupational; her husband was also a smoker, which was considered a confounding factor in determining causality. Coincidentally, the woman’s experience of injustice was not as articulated as that of Maria and her family. She knew that her husband’s illness very likely resulted from asbestos exposure, yet her experience with the compensation system did not give her the legitimacy nor the language to advocate for justice:

After all this time, my daughter and I are still upset about this. We applied a few times for compensation, but always got rejected… It was a disappointment, not in the sense that we wanted the money, because that money is the money of a person who is no longer here, it’s not a nice thing… it would have just been good to know that his illness was recognized, because otherwise you are left with the doubt.

The ‘doubt’ that the woman described goes back to the uncertainty that people in Taranto experience with illness and shapeshifting pollutants. The failed attempt to gain legitimacy over one’s disease prevents workers and residents alike from navigating a bureaucratic system that provides a language to articulate suffering and seek justice. Below, I focus on how the bureaucratic toxicity of asbestos has leaked into the experience of diseases that are correlated to shapeshifting pollutants, creating the assumption that if bureaucratic pathways to access justice are not predetermined, then justice cannot be fully pursued.

### Toxic flows and slow observation

The ubiquity of asbestos, its experiential detachment from illness and the bureaucratization of experiences of exposure, illness, death and justice lead to a naturalization of the substance in what can be called a toxic nature (Lora-Wainwright, 2017) for the community. With this vivid narrative informing the looser bureaucracy of shapeshifting pollutants, illness is crystallized as inevitable, leading to the emergence of a general resignation that amplifies the harm to public health. As the experience of asbestos is validated through bureaucracy, some of its specific cognitive and experiential characteristics are also imprinted on other pollutants and illnesses.

An example of the approach to toxicity in Taranto is illustrated by an encounter I had with a worker at his home. As we were talking about air pollution in the Tamburi district, he mentioned that when the wind blows from north–west, the dust from the mineral parks of the factory covers the rooftop of his house. People in Taranto are aware that the mineral dust is extremely toxic and that they should minimize exposure by avoiding skin contact and inhalation. However, when we reached the rooftop of his building, this informant did not hesitate to collect some of it with his bare hand so he could show it to me. When I reminded him that dust is not meant to be touched, he responded that he was perfectly aware of it. He explained that, from his perspective, dust had become such a constitutive element of windy days that being exposed to it was perfectly natural. He recalled that dust had been on rooftops since he was a child, even when industrial pollution was not a prominent part of social discourse. Given this unofficial long-term exposure, touching the dust does not alter his likelihood of getting sick in the future. This understanding of the pollutant was interestingly similar to the approach to asbestos discussed by other workers.

These similarities between asbestos and shapeshifting pollutants also emerged in other interviews, particularly with reference to illness, which was perceived as inevitable and almost independent from exposure. This temporal dissonance between exposure, disease onset and death is accompanied by a sense of inevitability, with suffering not being translated into injustice but accepted as natural. Residents seem to have fully acknowledged that their past exposure inhabits their future – what Fortun (2001) calls future anterior¸ ‘like the way toxics inhabit the bodies of those exposed, setting up the future, but not yet manifest as disease’. When discussing the theme of cancer with a worker, he said:

[cancer] can stay there for years and years, then one day it decides it must kill you and it becomes active. And then you’re dead. It might be that I even have one right now, I don’t know - touch wood! One time a colleague of mine managed to retire in September, only to pass away in October. […] this makes you think a lot of things, that at the end of the day we should be more humane when we’re still alive. It makes you think that it’s pointless to worry and argue and fight… in the end we are no one. You think you can do anything, and the next day you’re dead. You are less than nothing.

The interviews collected showed that, with inexistent legal pathways recognizing exposure and providing the means to act upon it, the workers and residents of the most polluted districts experience difficulty in recognizing the injustice of pollution and mobilizing. This produces a sort of resignation toward death that has been observed in other highly polluted communities from lower socio-economic backgrounds (Auyero and Swistun, 2009; Lora-Wainwright, 2017). Another retired worker commented:

[…] of course people are angry but then they stop for a second and think that thanks to Ilva they could feed a whole family! […] anyways everybody knows that we are all going to die of cancer, we are completely aware of this by now. I have to thank God for being well today, because I do not lack anything, I have a good pension… that’s it!

With illness and death being inevitable, workers and residents alike rather decide to focus on the positive aspects of their lives. While, on the one hand, this produces a sort of political detachment from the issue of exposure, on the other hand it allows the workers to create a space in which a new meaningfulness can be attributed to their identity as exposed workers and citizens: people that, through their hard work, can enjoy the serenity of a modern life (see Lora-Wainwright, 2013).

In other cases, further burdens are added to the widespread sense of powerlessness in the community: because shapeshifting pollutants can always be contested, the factory is also able to rewrite the source of toxic substances and shift liability on the sufferers themselves, as explained by this farmer who lives near the factory:

My mom had passed away because of a tumor. I also had surgery because of a tumor when I was 30, and got a kidney taken out… So in terms of health, we had already paid enough […] some experts even said that Ilva had nothing to do with this, that it was the farmers’ fault, that who knows what [chemical] we had used, they were kind of blaming it all on us.

The corporate legal capacity and the political connections of the factory grant it great bureaucratic power over pollutants, which are shaped to guarantee or increment productivity, as shown by the Save-Ilva decrees. Mirroring the experience of the farmer, between 2008 and 2013, spokespeople from the government and industry argued on several occasions that the scientific evidence gathered by civil society was not sufficient to support their claims against the factory. Power imbalances between residents and the industrial establishment are also reproduced in bureaucracy at different speeds, with the central government able to change laws in days and the residents subjected to the slower bureaucratic time of queues for medical checks, paperwork being processed, complaints being filed, delayed reports by public authorities, etc. However, if this practice of waiting is a form of power exerted over the community (Auyero and Swistun, 2009), it also represents a hidden space where the exposed learn to make sense of contamination over extended periods of time, eventually leading to political consideration and potential intervention.

A closer look at Taranto’s experience of pollutants expands the notion of slow observation (Davies, 2018) to include the lived micro interactions with legal and bureaucratic systems that give people a voice to articulate their suffering. Within this temporal scale, toxicity does not follow the time of epidemiology but flows through the legal infrastructure that (in)validates evidence. The constant oscillation of the parameters of acceptability of toxicity entails the formation of new experiential paradigms that will require significantly extended periods of time to change. Today, the narrative of asbestos exposure that framed the experience of toxicity in two generations of workers is slowly shifting through the legal victories gained by environmental organizations after more than a decade.

## Conclusion

At the time of writing this article, Taranto’s trial for environmental disaster started with the 2012 investigations is coming to an end. Much to the surprise of activists, the public prosecutor on the trial has taken an adamant position on ex-Ilva, accusing the 47 defendants of ‘criminal’ management of the factory, and stating that most self-monitored data on environmental emissions were consistently ‘falsified’ between 1995 and 2013. The developments come only a few weeks after the results of a 2014 investigation on the death of a 5-year-old to brain cancer. For the first time in the history of Taranto, the public investigation formally recognized causality between exposure to chemicals in the air and the death of the child, setting up a precedent that will inevitably shape the course of law in future investigations. Although these developments are still too recent to draw conclusions on the future of Taranto, many environmental activists are hopeful that things are beginning to change. They believe that the general population, too, is paying more attention to pollution, with injustice being more visible and embedded into the residents’ lived experience. Alessandro smiles and says with contentment: ‘we have been saying this for over 10 years - finally people are starting to see’.

In conclusion, a closer look at toxic flows in Taranto shows that the toxicities that matter to environmental health do not necessarily coincide with those that shape the lived experience of residents. As demonstrated, the political experience of shapeshifting pollutants in Taranto has been a terrain widely unregulated by weak institutions and slow bureaucracy. The absence of a legal framework directly recognizing the damage to the community is paired with special laws that often prevent the community from mobilizing epidemiological evidence and advocate for environmental justice. The workers’ experience of asbestos here represented an exception to this wider category of unregulated pollutants. The institutional exceptionality granted to asbestos has imbued the pollutant with a high symbolic power that shapes the community’s cultural and political experience of pollution.

In the context of this special issue, this paper contributes by shedding light on the bureaucratic and temporal dimensions of toxic flows. Embedded within public discourse in the city, pollutants flow beyond the epidemiological scale of environmental disaster and are re-evaluated through the slow lived experience of legal and bureaucratic apparatuses. Attuning to these temporalities through slow observation can disclose experiential dissonances that prevent some of the workers and residents from taking part in the political life of the community. Aside from law and bureaucracy, toxic flows pervade similarly unexpected corners of life, becoming responsible for new configurations of resignation and alternate aspirations to environmental justice. A more rigorous questioning of these dissonances is required in order to shed light on unconforming epistemologies of environmental justice.

## Notes

1 The ongoing trial, also known as Ambiente Svenduto (Sold Off Environment), sees accusations against 47 defendants (44 persons and three societies) that were involved in the management of the factory between 1995 and 2013.

2 Direct exposure on the workplace was recognized up until 1992, but indirect exposure has continued in some cases until the termination of the reclamation works on 2 October 2003. – https://www.inps.it/nuovoportaleinps/default.aspx?itemdir=46078

## References

[bibr1-23996544221107517] Auyero J Swistun DA (2009) Flammable: Environmental Suffering in an Argentine Shantytown. New York, NY; Oxford: Oxford University Press.

[bibr2-23996544221107517] Banini T Palagiano C (2014) Environment and health in italian cities: the case of taranto, pp. 17–37.Environmental Deterioration and Human Health: Natural and Anthropogenic Determinants.

[bibr3-23996544221107517] Barca S Leonardi E (2016) Working-class communities and ecology: reframing environmental justice around the Ilva steel plant in Taranto (Apulia, Italy). Policy Press. Class, Inequality and Community Development.

[bibr4-23996544221107517] Barca S Leonardi E (2018) Working-class ecology and union politics: a conceptual topology. Globalizations 15(4): 487–503.

[bibr5-23996544221107517] Barnett C (2016) Sing along with the Common People”: Politics, Postcolonialism, and other Figures: Environment and Planning D: Society and Space. London, England: SAGE PublicationsSage UK.

[bibr6-23996544221107517] Bingham N (2008) Slowing things down: lessons from the GM controversy. Geoforum 39(1): 111–122.

[bibr7-23996544221107517] Bismillah AN Chapin BM Hussein BA , et al. (2020) Shapeshifting molecules: the story so far and the shape of things to come. Chemical Science The Royal Society of Chemistry, 11(2): 324–332. 10.1039/c9sc05482k PMC7069523 32206269

[bibr8-23996544221107517] Brown P (2007) Toxic Exposures: Contested Illnesses and the Environmental Health Movement. Columbia University Press.

[bibr9-23996544221107517] Checker M (2007) ‘But i know it’s true’: environmental risk assessment, justice, and anthropology. Human Organization 66(2): 112–124.

[bibr10-23996544221107517] Congedo A (2017) Amianto: i dati di Taranto fanno impallidire quelli del resto d’Italia. Inchiostro Verde. Available at: http://www.inchiostroverde.it/2017/07/03/63176-2/(accessed 26 May 2020).

[bibr11-23996544221107517] Davies T (2018) Toxic space and time: slow violence, necropolitics, and petrochemical pollution. Annals of the American Association of Geographers 108(6): 1537–1553.

[bibr12-23996544221107517] Davies T (2019) Slow violence and toxic geographies: ‘Out of sight’ to whom? Environment and Planning C: Politics and Space, 2399654419841063.

[bibr13-23996544221107517] Davies T Mah A (eds), (2020). Toxic Truths: Environmental Justice and Citizen Science in a Post-Truth Age. S.l, Manchester University Press.

[bibr14-23996544221107517] Fortun K (2001) Advocacy after Bhopal: Environmentalism, Disaster, New Global Orders. Chicago ; London: University of Chicago Press.

[bibr15-23996544221107517] Gomellini M Toniolo G (2017) The Industrialization of Italy, 1861–1971. In: The Spread of Modern Industry to the Periphery since 1871, Oxford: Oxford University Press.

[bibr16-23996544221107517] Greco C (2016) Blaming the southern victim: cancer and the Italian ‘Southern Question’ in Terra dei fuochi and Taranto. Anthropology Today 32(3): 16–19.

[bibr17-23996544221107517] Gullion JS (2015) Fracking the Neighborhood: Reluctant Activists and Natural Gas Drilling. Cambridge, MA: The MIT Press.

[bibr18-23996544221107517] Hatzisavvidou S (2015) Disturbing binaries in political thought: silence as political activism. Social Movement Studies 14(5): 509–522.

[bibr19-23996544221107517] Ippolito, AR (2022) Beyond Protest – The Moral Struggle of Industrial Pollution in Taranto, Italy.. In: Bonneuil C (ed), In the Shadow of the Petrochemical Smokestack - Chemical Corridors and Environmental Health. Paris: Le Seuil. Available from: https://www.researchgate.net/publication/361143142BeyondProtest-TheMoralStruggleofIndustrialPollutioninTarantoItaly (Accessed 07 June 2022).

[bibr20-23996544221107517] Jungkunz V (2012) The promise of democratic silences new political science 34(2): 127–150.

[bibr21-23996544221107517] Kleinman MDA (2008) What Really Matters: Living a Moral Life amidst Uncertainty and Danger. Oxford, NY: Oxford University Press.

[bibr22-23996544221107517] Lerner S (2005) Diamond: A Struggle For Environmental Justice in Louisiana’s Chemical Corridor. 1st ed. Cambridge, MA: Mit Pr.

[bibr23-23996544221107517] Lora-Wainwright A (2013) Fighting for Breath: Living Morally and Dying of Cancer in a Chinese Village. University of Hawai’i Press.

[bibr24-23996544221107517] Lora-Wainwright A (2017) Resigned Activism: Living with Pollution in Rural China. Mit Press.

[bibr25-23996544221107517] Mangia C Gianicolo EAL Bruni A , et al. (2013) Spatial variability of air pollutants in the city of Taranto, Italy and its potential impact on exposure assessment. Environmental Monitoring and Assessment 185(2): 1719–1735. 22585403 10.1007/s10661-012-2663-4

[bibr26-23996544221107517] Mataloni F Stafoggia M Alessandrini E , et al. (2012) A cohort study on mortality and morbidity in the area of Taranto, Southern Italy. Epidemiologia E Prevenzione 36(5): 237–252. 23139110

[bibr27-23996544221107517] Mazzeo A (2018) The temporalities of asbestos mining and community activism. The Extractive Industries and Society 5(2): 223–229.

[bibr28-23996544221107517] Mbembe A (2019) Necropolitics. Durham: Duke University Press Books.

[bibr29-23996544221107517] Mountz A Bonds A Mansfield B , et al. (2015) For slow scholarship: a feminist politics of resistance through collective action in the neoliberal university. ACME, International E-Journal for Critical Geographies.

[bibr30-23996544221107517] Nixon R (2011) Slow Violence and the Environmentalism of the Poor. Cambridge, MassLondon: Harvard University Press.

[bibr31-23996544221107517] Paglietti F Malinconico S Molfetta VD , et al. (2012) Guidelines for asbestos remediation at italian superfund sites. Journal of Environmental Science and Health, Part C. 30(3): 253–286. 10.1080/10590501.2012.705161 22970721

[bibr32-23996544221107517] Pass H Giroux D Kennedy C , et al. (2016) The IASLC mesothelioma staging project: improving staging of a rare disease through international participation. Journal of Thoracic Oncology: Official Publication of the International Association for the Study of Lung Cancer, 11(12). 10.1016/j.jtho.2016.09.123 27670823

[bibr33-23996544221107517] Pennuzzi M (2001) Storia dello stabilimento dalla nascita ai giorni nostri. Materiale Centro Studi CGIL Taranto.

[bibr34-23996544221107517] Petryna A (2013) Life Exposed: Biological Citizens after Chernobyl. With a New introduction. Princeton, NJ: Princeton University Press.

[bibr35-23996544221107517] Pirastu R Ancona C Iavarone I , et al. (2010) SENTIERI Project. Mortality study of residents in Italian polluted sites: evaluation of the epidemiological evidence. Epidemiologia E Prevenzione 34(5–6 Suppl 3): 1–2. 21220828

[bibr36-23996544221107517] Shapiro N (2015) Attuning to the chemosphere: domestic formaldehyde, bodily reasoning, and the chemical sublime. Cultural Anthropology 30(3): 368–393.

[bibr37-23996544221107517] Stengers I (2018) Another Science Is Possible: A Manifesto for Slow Science (tran. S Muecke). 1st ed. Cambridge ; Medford, MA: Polity.

[bibr38-23996544221107517] Vigotti MA Mataloni F Bruni A , et al. (2014) Mortality analysis by neighbourhood in a city with high levels of industrial air pollution. International Journal of Public Health 59(4): 645–653. 24760197 10.1007/s00038-014-0554-x

[bibr39-23996544221107517] Vulpio C (2012) La città delle nuvole: Viaggio nel territorio più inquinato d’Europa. Edizioni Ambiente.

[bibr40-23996544221107517] Waldman L (2009) ‘Show me the evidence’: mobilisation, citizenship and risk in indian asbestos issues. IDS Working Papers 2009(329): 01–48.

[bibr41-23996544221107517] Whatmore SJ (2009) Mapping knowledge controversies: science, democracy and the redistribution of expertise. Public Health Genomics 33(5): 587–598.

